# Supplementary material for: Daily variation in blood glucose levels during continuous enteral nutrition in patients on the intensive care unit: a retrospective observational study
Source: eBioMedicine. 2024 May 30;104:105169. doi: 10.1016/j.ebiom.2024.105169 (PMC11177052; doi:10.1016/j.ebiom.2024.105169)
Supplement: Supplementary Methods S1 and S2, Figs. S1–S6, and Tables S1–S5 [file mmc1.pdf]

Supplementary Materials

Daily variation in blood glucose levels during continuous enteral nutrition in patients on the Intensive Care Unit: a retrospective observational study

Authors: F.W. Hiemstra[1,2], D.J. Stenvers[3,4,5], A. Kalsbeek[3,5,6,7], E. de Jonge[1], D.J. van Westerloo[1], L. Kervezee[2]

[1]Department of Intensive Care, Leiden University Medical Center, Albinusdreef 2, 2333 ZA, Leiden, The Netherlands
[2]Group of Circadian Medicine, Department of Cell and Chemical Biology, Leiden University Medical Center, Albinusdreef 2, 2333 ZA, Leiden, The Netherlands
[3]Department of Endocrinology and Metabolism, Amsterdam UMC location University of Amsterdam, Meibergdreef 9, 1105 AZ Amsterdam, The Netherland[4]Department of Endocrinology and Metabolism, Amsterdam UMC location Vrije Universiteit, Meibergdreef 9, 1105 AZ Amsterdam,, The Netherlands
[5]Amsterdam Gastroenterology Endocrinology Metabolism, Amsterdam UMC, Meibergdreef 9, 1105 AZ Amsterdam, The Netherlands
[6]Netherlands Institute for Neuroscience (NIN), Royal Dutch Academy of Arts and Sciences (KNAW), Meibergdreef 47, 1105 BA Amsterdam, The Netherlands
[7]Laboratory of Endocrinology, Department of Laboratory Medicine, Amsterdam UMC location University of Amsterdam, Meibergdreef 9, 1105 AZ Amsterdam, The Netherlands

TABLE OF CONTENTS

SUPPLEMENTARY METHODS 3

Supplementary Methods S1: XGBoost analysis using SHAP values.
Supplementary Methods S2: Formulas of linear mixed-effects models.

SUPPLEMENTARY TABLES 4

Supplementary Table S1: Carbohydrate content for each nutrition product present in the dataset.
Supplementary Table S2: Overview of variables included in the linear mixed-effects models and XGBoost model.
Supplementary Table S3: Characteristics of included glucose measuring (during enteral feeding episodes).
Supplementary Table S4: Results of log-likelihood ratio test to compare model fits.
Supplementary Table S5: Number of glucose measurements and patients for each subgroup in sensitivity analysis.

SUPPLEMENTARY FIGURES 7

Supplementary Figure S1: Overview of variables included in the statistical analysis.
Supplementary Figure S2: Proportion of glucose measurements taken during administration of insulin, dextrose, and glucocorticoids by time of day.
Supplementary Figure S3: Glucose levels over time, normalised per patient.
Supplementary Figure S4: Model diagnostic plots of final linear mixed-effects model.
Supplementary Figure S5: SHAP analysis of XGBoost regression model.
Supplementary Figure S6: SHAP dependence plots of carbohydrate administration rate, insulin administration rate, dextrose administration rate and age.

REFERENCES 13

SUPPLEMENTARY METHODS

Supplementary Methods S1: XGBoost analysis using SHAP values.

In order to show robustness of our results obtained with the linear mixed-effects model, the analysis was repeated using the same dataset with an Extreme Gradient Boosting (XGBoost) regression model.[1] XGBoost is a widely adopted machine learning algorithm that uses ensemble learning of decision trees within a gradient boosting framework and is known for its computational efficiency and good performance. XGBoost analysis and visualization was performed using Python programming language (version 3.10.2) using xgboost package version 1.7.6[1], shap package version 0.41.0[2] and scikit-learn version 1.3.0.[3]

The variables that were used as fixed effects in linear mixed-effects model 4 were used as features in a XGBoost regression model: age, sex, diabetes diagnosis, time of day, administration rates of carbohydrate (grams/hour), dextrose (grams/hour) and insulin (units/hour) and administration of glucocorticoids (yes/no). In contrast to linear mixed-effects model, time of day was used as continuous variable (in decimal units from 0 to 24) in the XGBoost regression model (ranging from 0 to 24), and age and administration rates of carbohydrate, insulin and dextrose were used as continuous variables. Glucose level (in mmol/L) was the target variable (Supplementary Table S1).

Tuning of hyperparameter (i.e. a parameter that is set before the learning process and whose value is used to control the learning process) is essential to optimise model performance and generalization. We used a grid search with 5-fold cross-validation for hyperparameter tuning. The ranges of hyperparameters for the grid search were as follows: 1) Maximum tree depth: 5, 10, 15, 25, 50; 2) Learning rate: 0·05, 0·1, 0·3; 3) Number of estimators: 25, 50, 100, 150; 4) Minimum child weight: 1, 5, 10; 5) Fraction of columns to be subsampled in each tree; 0·75, 1·0; 6) Subsample ratio of training instances: 0·75, 1·0. Default settings of the xgboost Python package were used for the rest of the hyperparameters.[1]

In order to assess the effect of time of day (and the other variables included as features in the model) on glucose levels, the SHapley Additive exPlanations (SHAP) method was used.[2] SHAP is a method used in machine learning to explain how each feature contributes to the model's prediction. It assigns importance scores (SHAP values) to each feature for each single observation (which is in our study a glucose measurement) when considered in combination with all features in the model. The magnitude of the SHAP value thus represents the increase or decrease in the glucose level that can be attributed to the feature value.

Supplementary Methods S2: Formulas of linear mixed-effects models.

Model	Formula (*lme4* R notation)
**Model 1**	`glucose_level ~ 1 + (1 | subjectID)`
**Model 2**	`glucose_level ~ diabetes + age + sex + (1 | subjectID)`
**Model 3**	`glucose_level ~ diabetes + age + sex + carbohydrate_administration_rate + insulin_administration_rate + insulin_administration_rate + glucocorticoid_administration + (1 | subjectID)`
**Model 4**	`glucose_level ~ diabetes + age + sex + carbohydrate_administration_rate + insulin_administration_rate + insulin_administration_rate + glucocorticoid_administration + time_of_day (1 | subjectID)`

SUPPLEMENTARY TABLES

Supplementary Table S1: Carbohydrate content for each nutrition product present in the dataset.

Nutrition type	Carbohydrate content	Source
**Beneprotein**	0	https://www.nestlehealthscience.ca/en/brands/beneprotein/beneprotein-hcp
**Boost Glucose Control**	0.068	https://www.boost.com/products/glucose-control
**Enlive**	0.190	Fitgerald et al. (2021)[4]
**Ensure**	0.139	Fitgerald et al. (2021)[4]
**Ensure Plus**	0.203	Fitgerald et al. (2021)[4]
**Fiber Supplement (i.e. Banana Flakes)**	NOT FOUND	N/A
**Fibersource HN**	0.156	Fitgerald et al. (2021)[4]
**Glucerna**	0.11	Fitgerald et al. (2021)[4]
**Impact**	0.132	Fitgerald et al. (2021)[4]
**Impact with Fiber**	0.132	Fitgerald et al. (2021)[4]
**Isosource 1.5**	0.167	Fitgerald et al. (2021)[4]
**Jevity 1.2**	0.170	https://www.abbottnutrition.com/our-products/jevity-1_2-cal
**Jevity 1.5**	0.216	https://www.abbottnutrition.com/our-products/jevity-1_5-cal
**Nepro**	0.147	Fitgerald et al. (2021)[4]
**NovaSource Renal**	0.185	Fitgerald et al. (2021)[4]
**Nutren 2.0**	0.196	Fitgerald et al. (2021)[4]
**Nutren Pulmonary**	0.10	Fitgerald et al. (2021)[4]
**Nutren Renal**	0.185	Fitgerald et al. (2021)[4]
**Osmolite 1.5**	0.203	Fitgerald et al. (2021)[4]
**Peptamen 1.5**	0.188	Fitgerald et al. (2021)[4]
**Peptamen Bariatric**	0.078	Fitgerald et al. (2021)[4]
**ProBalance**	NOT FOUND	N/A
**Promote**	0.131	https://www.abbottnutrition.com/our-products/promote
**Promote with Fiber**	0.139	https://www.abbottnutrition.com/our-products/promote-with-fiber
**Pulmocare**	0.105	Fitgerald et al. (2021)[4]
**Replete**	0.112	Fitgerald et al. (2021)[4]
**Replete with Fiber**	0.124	Fitgerald et al. (2021)[4]
**Two Cal HN**	0.219	Fitgerald et al. (2021)[4]
**Vital 1.5**	0.186	https://www.abbottnutrition.com/our-products/vital-1_5-cal
**Vital High Protein**	0.113	https://www.abbottnutrition.com/our-products/vital-hp
**Vivonex**	0.003	Fitgerald et al. (2021)[4]

Supplementary Table S2: Overview of variables included in the linear mixed-effects models and XGBoost model.

	Linear mixed-effects model						XGBoost model	
	**Variable type**	**Variable in model**	**Model 1**	**Model 2**	**Model 3**	**Model 4**	**Variable type**	**Variable in model**
**Glucose level**	Continuous	Dependent variable	X	X	X	X	Continuous	Target variable
**PatientID**	Categorical	Random effect	X	X	X	X		
**Intercept**			X	X	X	X		
**Patient level variables**								
**Age (years)**	Categorical [≤55, 55<x≤65, 65<x≤75 >75]	Fixed effect		X	X	X	Continuous	Feature
**Sex**	Categorical [female / male]	Fixed effect		X	X	X	Categorical [female, male]	Feature
**Diabetes diagnosis**	Categorical [yes, no]	Fixed effect		X	X	X	Categorical [yes, no]	Feature
**Sample level variables**								
**Carbohydrate administration rate (grams/hour)**	Categorical [≤4.5, 4.5<x≤6.5, 6.5<x≤8.5 >8.5]	Fixed effect			X	X	Continuous	Feature
**Insulin administration rate (units/hour)[a]**	Categorical [0, 0<x≤1.5, 1.5<x≤3, >3]	Fixed effect			X	X	Continuous	Feature
**Dextrose administration rate (grams/hour)[a]**	Categorical [0, 0<x≤0.5, 0.5<x≤2, >2]	Fixed effect			X	X	Continuous	Feature
**Glucocorticoid administration[a]**	Categorical [yes, no]	Fixed effect			X	X	Categorical [yes, no]	Feature
**Time variable**								
**Time of day**	Categorical [time bins 0 to 23]	Fixed effect				X	Continuous	Feature

[a] The start and/or end time of administration of insulin, dextrose and glucocorticoids were corrected to account for their delaying and/or persisting effect on glucose levels (see Methods for details).

Supplementary Table S3: Characteristics of included glucose measuring (during enteral feeding episodes).

Glucose measurement characteristics	Included glucose measurements (n=207,647)
**Glucose measurements per patient per day, median [IQR]**	3 [2-5]
**Glucose measurements taken during administration of:, n (%)**	
**Dextrose[a]**	89,581 (43%)
**Insulin[a]**	70,066 (34%)
**Glucocorticoids[a]**	40,314 (19%)
**Sample type, n (%)**	
**Finger stick**	129,133 (62%)
**Lab (whole blood)**	64,489 (31%)
**Lab (serum)**	14,025 (6.8%)

[a] The start and/or end time of administration of insulin, dextrose and glucocorticoids were corrected to account for their delaying and/or persisting effect on glucose levels (see Methods for details).

Supplementary Table S4: Results of log-likelihood ratio test to compare model fits.

	Number of variables	Log-Likelihood	Degrees of freedom	Chi-squared value	P-value
**Model 1**	3	-484169			
**Model 2**	8	-483164	Compared to model 1		
			5	2009.5	<0.0001
**Model 3**	18	-479305	Compared to model 2		
			10	7719.3	<0.0001
**Model 4**	41	-478080	Compared to model 3		
			23	2449.1	<0.0001

Supplementary Table S5: Number of glucose measurements and patients for each subgroup in sensitivity analysis.

Variable	Number of glucose measurements	Number of patients
**Ventilation mode**		
**Non-Invasive**	20,408	2,416
**Invasive**	78,715	4,587
**Survivor status**		
**Non-survivors**	59,523	1,967
**Survivors**	148,124	4,962
**Sedation depth**		
**RASS ≥ -1**	96,191	5,191
**RASS ≤ -2**	81,903	4,532
**Day in the ICU**		
**Day 0-2**	14,714	3,251
**Day 3-7**	80,839	6,276
**Day ≥ 8**	112,094	3,718
**Sample type**		
**Point-of-care test**	129,133	6,199
**Lab test**	78,514	6,779
**Time to next glucose measurement**		
**< 4 hours**	91,845	5,689
**4-8 hours**	94,694	6,224
**> 8 hours**	18,108	4,621
**Insulin requirement**		
**No insulin requirement**	30,315	2,509
**Mean daily insulin administration of insulin:**		
**0<x<=35 units**	110,374	3,499
**35<x<=70 units**	24,692	460
**>70 units**	42,266	461

Note: Not all glucose measurements in the subgroups add up to 207,647 due to missing data.

SUPPLEMENTARY FIGURES

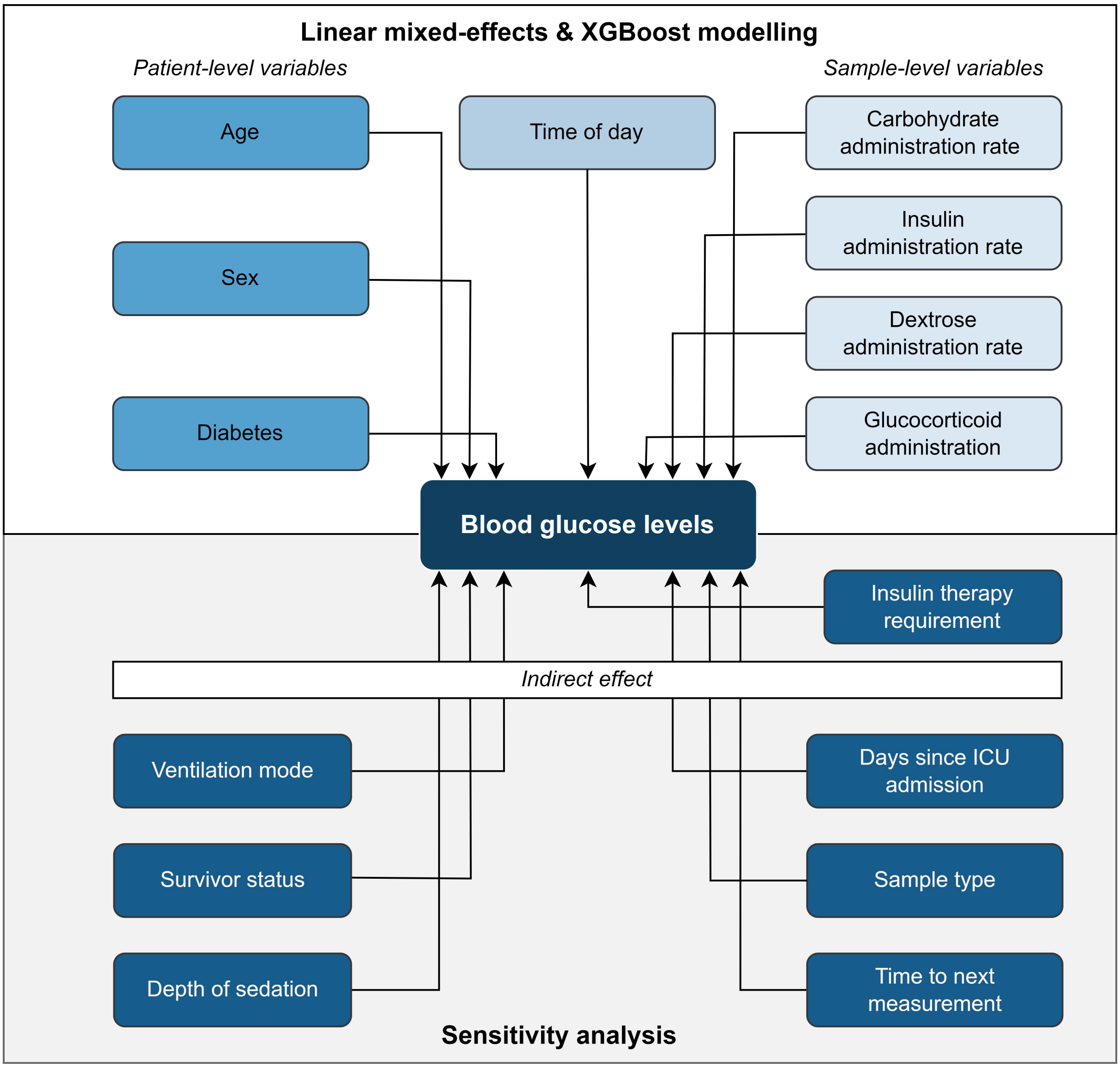

Supplementary Figure S1: Overview of variables included in the statistical analysis. Variables in the upper half of the figure are included in the linear mixed-effects models and XGBoost model. The variable groups (patient-level variables and sample-level variables) that are sequentially used in the various linear-mixed effects models (model 1-4) are represented in the figure. Variables in lower half of the figure are used to define the subgroups that are assessed in the sensitivity analyses.

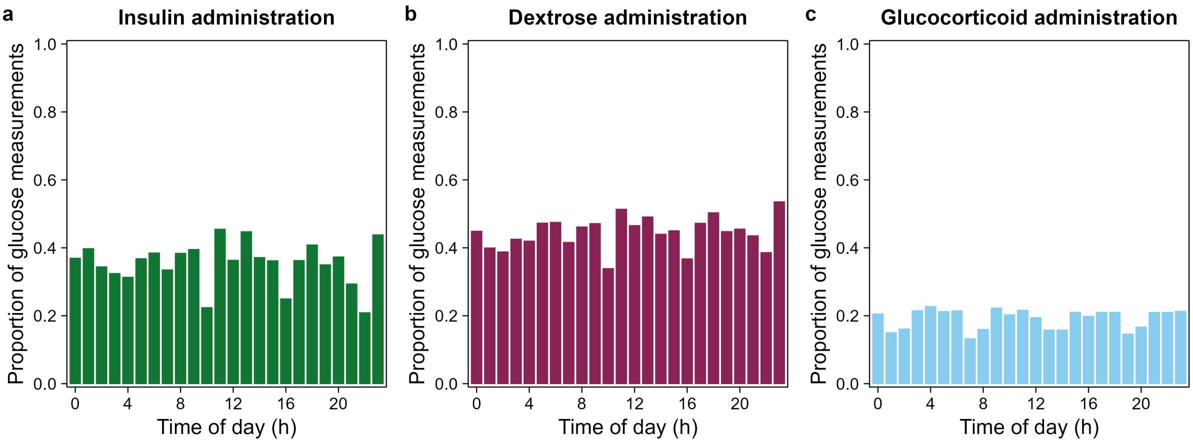

Supplementary Figure S2: Proportion of glucose measurements taken during administration of (a) insulin, (b) dextrose, and (c) glucocorticoids by time of day. The start and end time of administration of insulin and glucocorticoids (end times only) were corrected to account for the delaying and/or persisting effect of insulin and glucocorticoids on glucose levels (see Methods for details).

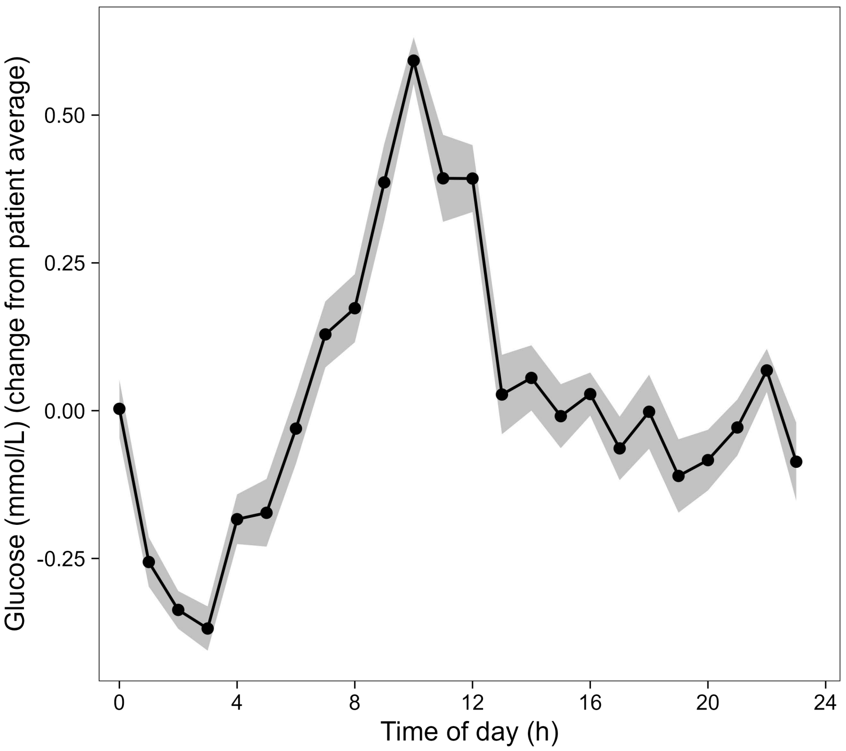

Supplementary Figure S3: Glucose levels over time, normalised per patient.

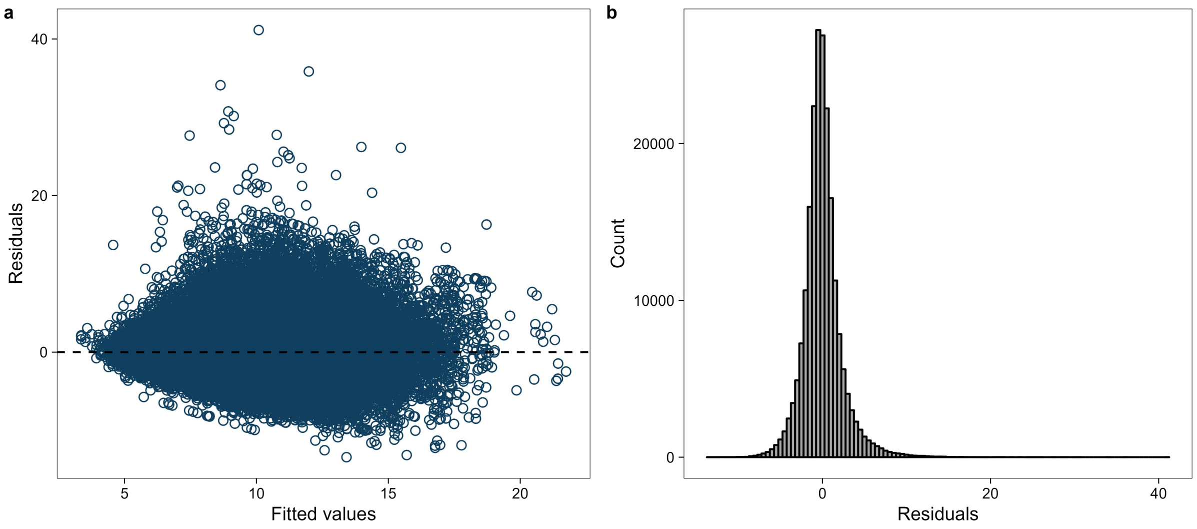

Supplementary Figure S4: Model diagnostic plots of final linear mixed-effects model. (a) Residuals versus fitted values. **(b)** Distribution of residuals.

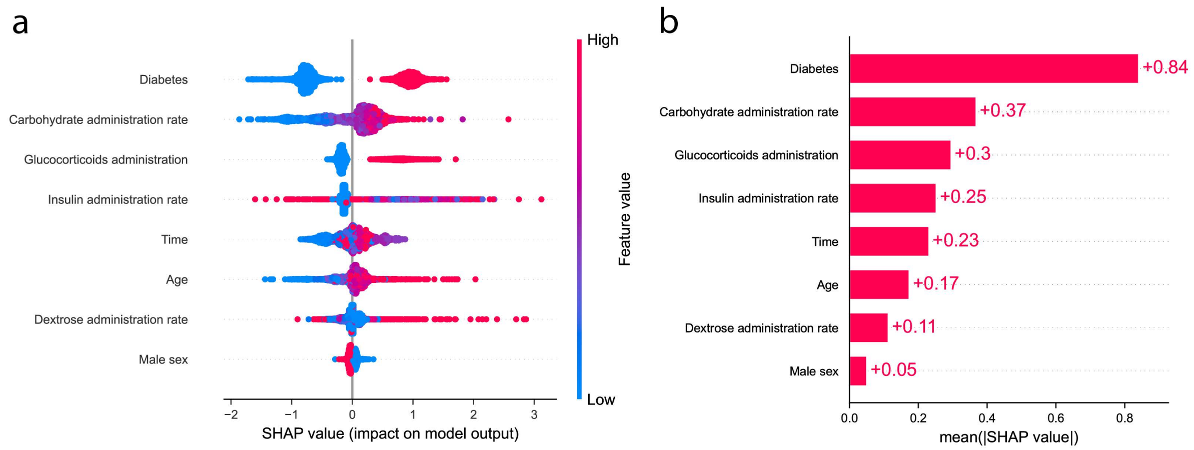

Supplementary Figure S5: SHAP analysis of XGBoost regression model. (a) SHAP Summary plot. Each dot represents an individual glucose measurement and is coloured according to the value of the feature. Red represents a higher feature value, blue represent a lower value. In categorical features, the red colour indicates the presence of the corresponding variable, while blue represents the absence. The horizontal location is of dot depicts whether it corresponds with a higher or lower prediction of the glucose levels (SHAP value). A sample of 5000 randomly selected features was used to in this SHAP analysis. **(b)** SHAP Global bar plots. For each feature, the global importance is indicated by taking the mean of absolute SHAP values (from panel a) for that feature over all selected samples.

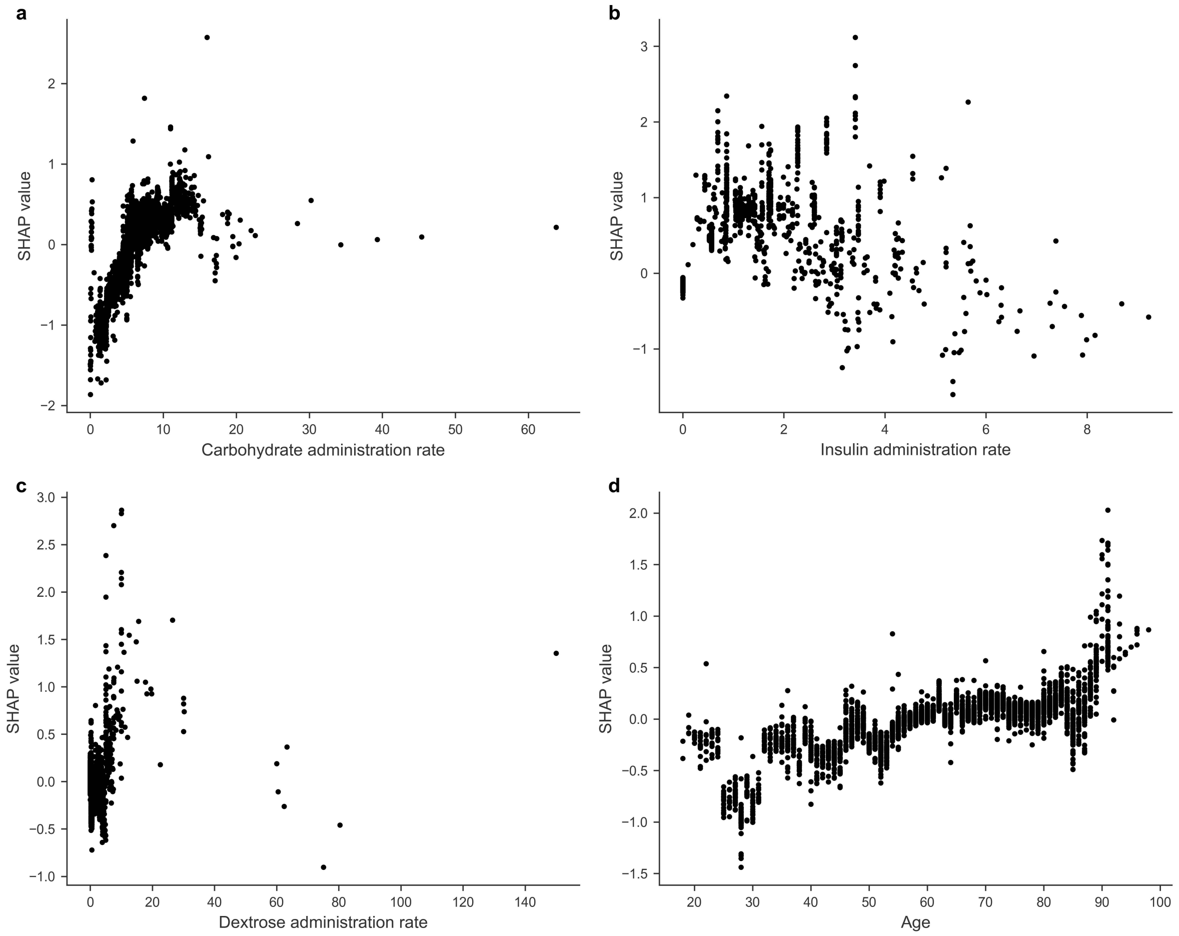

Supplementary Figure S6: SHAP dependence plots of (a) Carbohydrate administration rate (grams/hour), (b) Insulin administration rate (units/hour), (c) Dextrose administration rate (grams/hour) and (d) Age. Each dot represents an individual glucose measurement, with its feature value at the x-axis and its SHAP value at the y-axis. A sample of 5000 randomly selected features was used to in this SHAP analysis.

REFERENCES

1 Chen T, Guestrin C. XGBoost: A Scalable Tree Boosting System. Proceedings of the 22nd ACM SIGKDD International Conference on Knowledge Discovery and Data Mining; San Francisco, California, USA: Association for Computing Machinery; 2016. p. 785–94.

2 Lundberg SM, Erion G, Chen H, Degrave A, Prutkin JM, Nair B, et al. From local explanations to global understanding with explainable AI for trees. *Nature Machine Intelligence*. 2020;2(1):56-67.

3 Pedregosa F, Varoquaux G, Gramfort A, Michel V, Thirion B, Grisel O, et al. Scikit-learn: Machine Learning in Python. *J Mach Learn Res*. 2011;12:2825-30.

4 Fitzgerald O, Perez-Concha O, Gallego B, Saxena MK, Rudd L, Metke-Jimenez A, et al. Incorporating real-world evidence into the development of patient blood glucose prediction algorithms for the ICU. *Journal of the American Medical Informatics Association*. 2021;28(8):1642-50.
